# Supplementary material for: A carbon nanotube tape for serial-section electron microscopy of brain ultrastructure
Source: Nat Commun. 2018 Jan 30;9:437. doi: 10.1038/s41467-017-02768-7 (PMC5789869; doi:10.1038/s41467-017-02768-7)
Supplement: Supplementary file 1 — Supplementary Information [file 41467_2017_2768_MOESM1_ESM.pdf]

Supplementary Note 1. Drawbacks of carbon coated (cc)-Kapton tape

We evaluated the cc-Kapton tape quantitatively to understand how to meet the requirements of an ATUMtome tape. The surface of Kapton tape is hydrophobic and non-conductive, and shows severe charging during SEM imaging under high-vacuum conditions. Conductive carbon coating resolves this problem [1]. We embedded rat brain tissue in epoxy resin (Durcupan ACM, Sigma-Aldrich, St. Louis, U.S.A.) using a modified heavy metal staining (mHMS) histology protocol (Table 1) and obtained serial ultrathin sections of 50 nm-thickness using the ATUMtome (RMC Boeckeler, Tucson, AZ, U.S.A.). Unless otherwise stated, we used these sections for examination. We observed that the hydrophobic tape surface of cc-Kapton Tape (RMC Boeckeler, Tucson, AZ, U.S.A.) generates copious section wrinkles during section collection (Supplementary Fig. 1). This was overcome by hydrophilization of the tape surface by plasma discharge treatment (see below, Supplementary Fig. 1). Carbon coating of the tape was necessary to eliminate imaging-induced charging. However, we occasionally found significant charging problems affecting image quality, due to randomly occurring even lower conductance areas that we attributed to the variable thickness of the carbon coating, including continuously drifting images (Supplementary Fig. 1), as well as its relatively high and variant tape surface sheet resistance (19.2/107/6,530 $M\Omega\ \square^{-1}$ for three samples of cc-Kapton tape, RMC Boeckeler). In addition, we often found scratches on the tape surface, likely generated during the tape production process (Supplementary Fig. 1), which negatively impacted image data capture. Moreover, the coated carbon layer is partially lost with plasma discharge treatment (Supplementary Fig. 1). It was examined with a home-coated carbon Kapton tape of carbon evaporated 10 nm thick deposit layer which allowed us to measure the resistance for its relatively low sheet resistance (19.3 % reduction of conductivity; sheet resistance, 3367 ± 516 and 4172 ± 559 $\Omega\ \square^{-1}$ without and with plasma discharge treatment, respectively, n=3 each cc-Kapton tape; estimated conductance: 5.9 and 4.8 $S\ m^{-1}$, respectively). Alternatively, carbon coating can be done after section collection on plasma discharge treated carbon uncoated Kapton tape [2,3], though this is generally to be avoided due to signal loss and noise generation from the overlying carbon layer, which is especially pronounced when using in-lens secondary electron (SE) detection (In-lens SE) (Supplementary Fig. 1). These problems, and the fact that ready-to-use cc-Kapton tape is not supplied in a stable manner commercially, prompted us to search for a more optimal tape for ATUM. We

concluded that the cc-Kapton tape varied in quality and some might not provide good imaging conditions.

Supplementary Note 2. Examinations of potential tapes

To find the best replacement of the cc-Kapton tape for ATUM use, we tested many different tapes: copper foil, 8mm video tape, ITO (indium tin oxide) coated PET, germanium-coated PET, open-reel and CNT-coated PET tapes (Supplementary Fig. 2, Supplementary Table 1). We initially thought that the copper foil tape would be a good choice for the ATUM because of its low resistance ($0.1\ \Omega\ \square^{-1}$ for 20 μm thick copper foil). Indeed, we did not notice any charging problems during image acquisition, but the tape failed to remain flat when subsequently adhered to a silicon wafer for imaging (Supplementary Fig. 2). The uneven surface made it difficult to focus the microscope across serial sections. Next, ITO-coated PET tape was tested. ITO is a transparent metal conductor and is typically used for flat-panel displays and smart windows. We imaged an EM sample on ITO-coated tape with the In-lens SE and backscattered electron (BSE) detector (BSD). We found a stripe pattern in the images (Supplementary Fig. 2). The thin ITO layers on the tape surface likely affect the images as the endogenic signal. The stripe pattern noise was probably due to cracks of the ITO layer generated at the very small angle of the ATUMtome tape guide tip end (Supplementary Fig. 2). We also tested germanium-coated PET film and found the image was fuzzy, especially with the BSD (Supplementary Fig. 2). We concluded that the tape coated with the conductive metal substance is not suitable for thin section imaging with the SEM because its strong endogenic signals may interfere with images.

Supplementary Note 3. Open reel tape

We then tried the open-reel tape (Supplementary Fig. 2) for ultrathin section collection with the ATUM. We found that it had a good resilience for the tape guide of the ATUM (Supplementary Fig. 2) and was easy enough to handle for adherence to the flat surface of the wafer. We imaged the 50 nm-thick tissue sections on the open reel tape with In-lens SE and BSD with common acceleration voltage (2 keV and 5 keV, respectively). We found that the electron micrographs were full of bar-like magnetic particle noise (Supplementary Fig. 2). It clearly indicated that the open-reel tape has strong endogenous noise and is not suitable tape for ATUM.

Then, we realized that it would be advantageous to analyze electron interaction depth quantitatively. We tested the EM samples using the In-lens SE detector with different acceleration voltages (Sigma, Carl Zeiss Microscopy GmbH, Oberkochen, Germany) (Supplementary Fig. 9) to see whether the image would change with the different accelerating voltages. We found that the image without the bar-like magnetic particle noise could be obtained only using 1 keV, but images using 1.5 keV or more led to the incorporation of magnetic particle signals in the magnetic layer of the tape (Supplementary Fig. 9). It was due to the larger and deeper interaction volume at higher acceleration voltages, which was further confirmed using the simulation, Monte Carlo Simulation of electron trajectory in solids (CASINO)[4] (Supplementary Fig. 9). The SE is known to be generated on two occasions: at the primary electron incidence into the block (SE1) and upon the primary electrons back-scattering from the block surface (SE2) [5]. The SE1 may give information about the surface structure and the SE2 about the tissue block mainly. Therefore, the BSE trajectory line (red lines in Supplementary Fig. 9) may be the interaction volume for the generation of SE2. This indicates that it is essential to keep the electron interaction volume within the tissue section thickness (50 nm), because when the BSE interaction volume reaches the base tape beyond the tissue section, it incorporates the background endogenous noise from the tape. There are two ways to minimize this noise: either using an optimally low acceleration voltage for imaging to restrict the interaction volume depth or using a base tape with low intrinsic signal (low Z). To estimate the proportion of the signal from a brain section (50 nm-thick), we divided the number of BSEs reflected only from the section by the number of all BSEs including those from base tape, signal efficiency for a 50 nm-thick section (Supplementary Table 2). The values of signal efficiency for a 50 nm-thick section in different acceleration voltages reasonably indicated a proportion of the real signal from tissue contained in the image from the open reel tape (Supplementary Fig. 9). Therefore, we believe that the Monte Carlo simulation reproduced the images that incorporated the magnetic particle signals on the open-reel tape. These results indicate that imaging with an acceleration voltage that generates an appropriate interaction volume for a given section thickness should give the best image results.

To see the performance of BSD in our Sigma SEM, we also imaged the same section using BSD with different acceleration voltages (Supplementary Fig. 9). We found that images without magnetic particle noise could be obtained using 1-2 keV, and images

with 2.5 keV or more increasingly led to the incorporation of magnetic particle signals (Supplementary Fig. 9). This may indicate that the BSD was not capable of efficiently detecting the lower energy BSEs, which probably travel deeper in the tissue block and have likely lost their energy due to travel over the long distance. Therefore, the BSD tends to image the magnetic particle signals using higher acceleration voltage than with the In-lens SE, which is designed to detect low energy electrons very efficiently with the 'Beambooster' of the GEMINI column to accelerate the electrons with an additional 8 keV [6].

Supplementary Note 4. Properties of the CNT-coated PET tape

We examined SEM beam damage on the CNT tape to see the influence on imaging. Beam damage resistance was studied with direct beam radiation onto the tape surface with 6.4 μs dwell time, 3 nm pixel^{-1}, 4k x 4k image size, 8 mm working distance (wd), 60 μm aperture and high current mode at different acceleration voltages for imaging with the BSD. Using a side-mounted Everhart-Thornley detector (sET), which imaged mainly surface structure, and 3D laser scanning confocal microscope (SCM), which imaged surface structure in pseudo depth color (VX-250, Keyence Corporation, Osaka, Japan), we found that the tape showed depression damage, which was probably due to further crosslinking of the overcoat layer. The damage was well correlated with acceleration voltage strength and depression depth measured with the SCM (Supplementary Fig. 3). The PET film itself was stretched during manufacture to reduce deformation by heat, but the overcoat or hard coat layers were not. Therefore, the polymer used in the overcoat layer may generate additional cross-linkage with the heat from the SEM beam. On the other hand, we found that the cc-Kapton tape showed a slight expansion (Supplementary Fig. 3) and the expansion height was well correlated with acceleration voltage strength (Supplementary Fig. 3). The beam damage depression depth of the CNT tape was deep, especially for the high acceleration voltages, but only a fraction of electrons may reach the base tape beyond the 50 nm-thick tissue section. Therefore, we investigated the depression depth after imaging the tissue section.

We captured images of the 50 nm-thick tissue sections with varied acceleration voltage strengths and dwell times (Supplementary Figs. 4, 5) and analyzed the beam damage using the depression depth in order to understand the possible mechanism of the depression of the CNT tape and any influence on image quality. As we predicted, the

damage depression depth was much smaller than the ones directly on the tape (the depression depth of tissue section with BSD/the depression depth of CNT tape surface with BSD: average 53.2 ± 8.9%; the depression depth of tissue section with In-lens SE/the depression depth of CNT tape surface with BSD: average 63.5 ± 6.6% for 6 keV, 5 keV, 4 keV and 3 keV). We found that the depth was determined mainly by acceleration voltage strength significantly ($P<0.01$) (Supplementary Figs. 4, 5). The depression depth was quite similar between BSD and In-lens SE imaging, although the electron dose for the BSD imaging was about 5 times larger than that of the In-lens SE for the aperture size difference (60 μm vs 20 μm) (Supplementary Figs. 4, 5). There was only a faint depression using an acceleration voltage of 2 keV or less, 17 – 67 nm depth (Supplementary Figs. 4, 5). It seems probable that primary electrons generated the depression and the depth of the electrons was determined by the acceleration voltage strength, which was predicted by the Monte Carlo simulation, which was tuned to the images on the open reel tape (Supplementary Fig. 9, blue lines). The primary electrons likely produced further cross-linking of the over-coat polymer of the tape surface directly under the tissue section, as well as the epoxy resin where the tissue was embedded, causing the depression. Indeed, we also found similar, but smaller depression damage with the 50 nm-thick tissue section on cc-Kapton tape (data not shown), where the Kapton tape itself showed expansion beam damage (Supplementary Fig. 3). These results indicated that the beam damage depression depth could be little especially for imaging the tissue section on the CNT tape with low acceleration voltage.

Next, we determined whether the seam between tiled images was noticeable after stitching. We captured 2 x 2 tiled images with 3 nm $pixel^{-1}$, 3.2 μs dwell time, 4096 x 4096 pixel images at 7.3 mm working distance in 5 keV using BSD with our Sigma-Atlas5 SEM (Supplementary Fig. 8). Image stitching was easily and seamlessly processed (Supplementary Fig. 8). We verified that the depression damage by the electron beam during imaging did not cause subsequent problems in stitching.

Mechanical strength of the CNT tape was examined. The tape is stretched, bent and wetted during ATUM operation, so the tape for the ATUM should be mechanically strong. The CNT tape was known to be strong enough to withstand bending up to 180° with 2.3 mm radius of curvature (Supplementary Fig. 2), temperatures up to 85°C, humidity up to 85%, and high vacuum conditions. The CNT tape showed no change in its structure after passing through the water in the diamond knife boat during the

ATUMtome process. It showed no significant change over long term shelf storage, although PET in the base of the tape may become yellowish from ultraviolet irradiation. Therefore, it is better to keep it shaded. We believe that the CNT tape is sufficiently strong for ATUM use.

Chemical strength was examined, because tissue sections on the tape may be processed with histological procedures. We tested water, 0.05 M Tris-HCl buffered saline (TBS) with 0.1% of triton X-100, 1% uranyl acetate solution, lead citrate staining solution, antiserum, ethanol, isopropyl alcohol and plasma discharge treatment. The tape was unaltered, at least for imaging use.

We analyzed surface structure of the CNT tape. The tape surface has to be smooth and flat, so as not to influence the image. Using the BSD, In-lens SE and sET in Sigma SEM, we found that the CNT coating was relatively uniform (Supplementary Figs. 3, 6) and it did not show up at all in the tissue images of 50 nm-thick sections (Figs. 2a, b, 3, Supplementary Figs. 4, 5, 6). The CNTs are affixed on the entire tape surface to make uniform surface resistance across the entire tape length (Fig. 1b). Using the In-lens SE detector in the Sigma SEM, which captured the surface shape, only thick CNTs were visible on the surface of the tape (Supplementary Fig. 6). In SEMs with stage bias potential, which efficiently increases signals from shallow tissue depth [7,8], the CNTs were clearly visible on the tape surface using an In-lens SE detector (GeminiSEM 300/MultiSEM 505, Carl-Zeiss Microscopy GmbH, Oberkochen, Germany) (Supplementary Fig. 6), and in relatively low contrast using a BSD optimized for low acceleration voltage (OnPoint BSD, Gatan, Inc., Pleasanton, CA, U.S.A.) in the GeminiSEM 300 (Supplementary Fig. 6). The CNTs varied in diameter (10 – 40 nm) and some of which, especially thick ones, were shown through very thin tissue sections (≤ 25-30 nm thickness) or with 50 nm-thick plastic sections lacking brain tissue (not shown), but not through 50 nm-thick tissue sections (Supplementary Fig. 6). We concluded that the surface of the CNT tape was sufficiently smooth and flat for SEM imaging with thin sections (>30 nm-thick).

In contrast, the surface of the cc-Kapton tape was bumpy and scratches were frequently found (Supplementary Figs. 1, 3, 7). The irregular surface may be caused by non-uniform affixed carbon deposition. Particles (~10 nm diameter), which could be dirt produced during the pre-washing process with isopropanol of the Kapton tape, were

also attached to the tape surface (Supplementary Figs. 1, 3, 7), which may slightly affect images obtained with the In-lens SE, but not with the BSD (Supplementary Fig. 7).

Supplementary Methods

Simulation analysis

Monte Carlo simulations of electron trajectories for SEM imaging have been reported previously [47,68]. We simulated the electron trajectory for backscattered imaging in SEM with CASINO (ver. 2.48) [69], by using the atomic fraction value of epoxy resin (nH = 0.53, nC = 0.35, nO = 0.12 [68]) without metals used for staining process, because most of the brain tissue was composed of only epoxy resin without any stained membrane throughout the ultrathin section thickness. The electron beam energies were set at 1.0–6.0 keV with a step of 0.5 keV, and the beam radius was set at 10 nm. In the present simulation, 3,000 electron trajectories were displayed for each beam energy.

Statistics

We used Kruskal-Wallis test to compare beam damage depression depth of different acceleration voltage or dwell time conditions (Supplementary Figs. 4, 5).

Supplementary figures

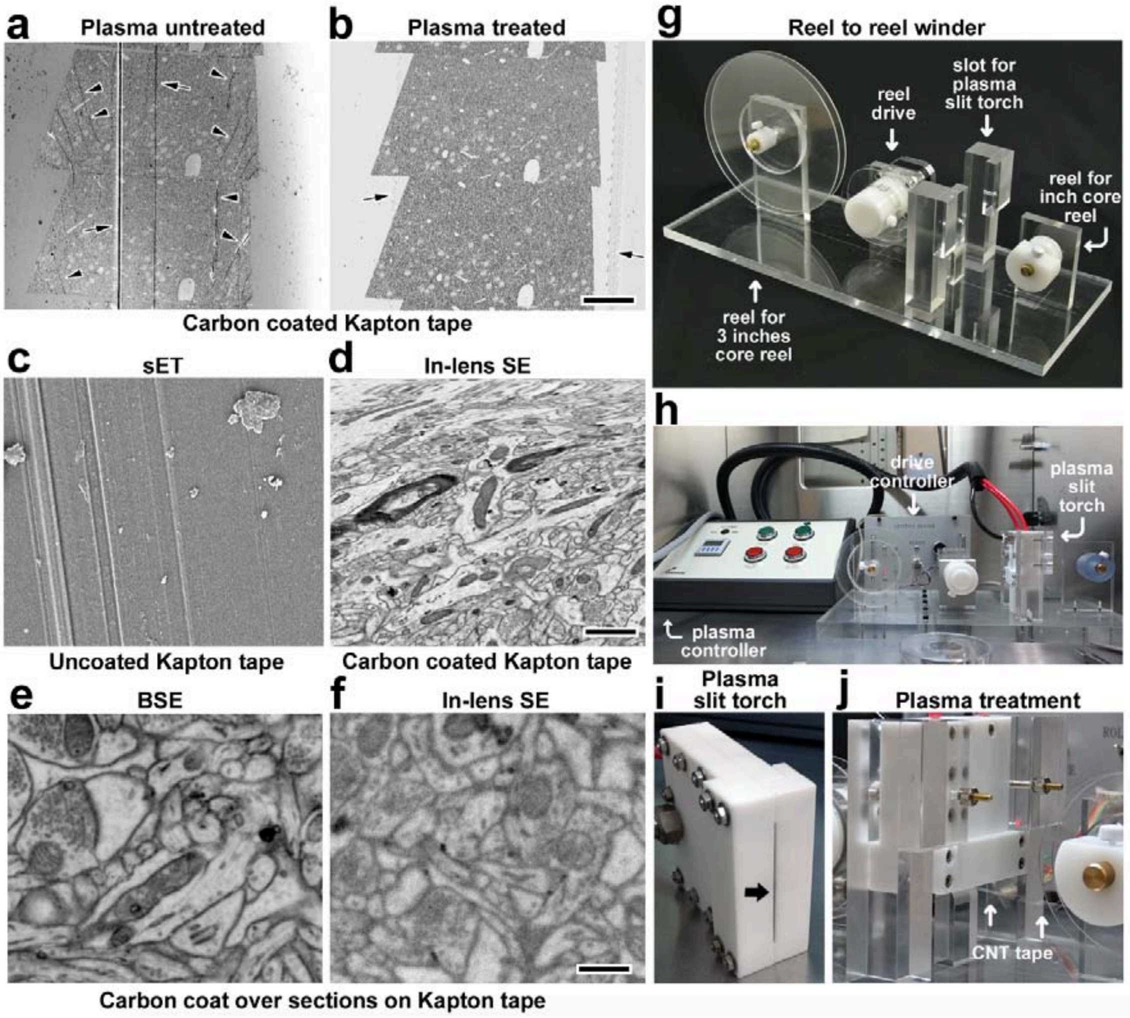

Supplementary Figure 1

Problematic features of Kapton tape and solutions using carbon coating and plasma discharge treatment

a. Non-plasma-treated cc-Kapton tape with collected serial ultrathin sections showing extensive wrinkles (arrow heads). Scratches on the tape surface were frequently seen (arrows), and they negatively impacted image data capture. **b**. Plasma-treated cc-Kapton tape containing ultrathin sections without wrinkles. Scratches on the tape surface were frequently seen (arrows). Scale, 100 μm, is also for (a). **c**. Scratches (arrows) are frequently seen on the surface of uncoated Kapton tape. **d**. Image obtained with a BSD generates continuously drifting images (evident in the upper part) due to charging. Scale, 2 μm, is also for (c). **e, f**. Images with high magnification of a tissue section on Kapton

tape with a carbon layer deposited on top of the section shows good ultrastructure with the BSD (**e**) and a fuzzy image taken with the In-lens SE detector (**f**). Scale, 0.5 μm, is also for (**e**). **g**. The reel-to-reel motorized winder for atmospheric pressure plasma glow discharge treatment. **h**. The atmospheric pressure plasma glow discharge system. **i**. Bottom view of the plasma slit torch head. Glowing plasma discharge shines through the center of narrow slit (arrow). **j**. Plasma slit torch head and the CNT tape (arrows).

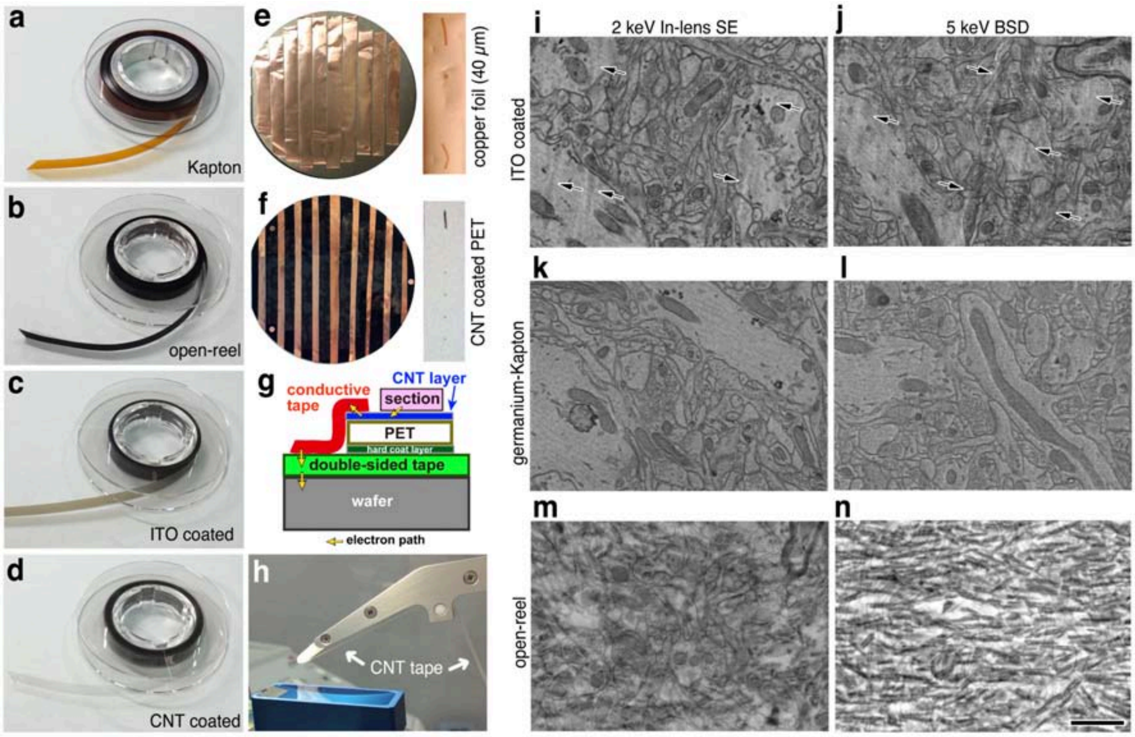

Supplementary Figure 2

Wide variety of tapes examined for ATUM

a. Kapton tape. **b**. open-reel tape. **c**. ITO-coated PET tape. **d**. CNT-coated PET tape. **e**. Serial ultrathin sections on strips of copper foil tape glued on a 4-inch silicon wafer. Ribbons of the serial ultrathin sections from a tissue block on copper foil (right). **f**. Serial ultrathin sections on strips of the CNT-coated PET tape (black strips) glued on a 4-inch silicon wafer. Conductive copper foil tape thin strips are adhered between the CNT-coated PET tape to ground the CNT layer to the wafer. Serial ultrathin sections collected on the CNT tape (right). **g**. Schematic drawing shows side view of the CNT tape with tissue section on the wafer. Possible electron pathway from the tissue section to wafer via the conductive CNT tape (yellow arrows). **h**. Tape guide tip of the ATUMtome. **i-n**, Images of mHMS-treated brain tissue captured with the In-lens SE detector at 2 keV (**i, k, m**), or the BSD at 5 keV (**j, l, n**) on ITO-coated PET tape (**i, j**), germanium-coated tape (**k, l**), or open-reel tape (**m, n**). Cracks of the ITO layer generated at the ATUMtome tape guide tip end are indicated by arrows in **i, j**. Scale in (**n**), 1 μm, is also for (**i-l**).

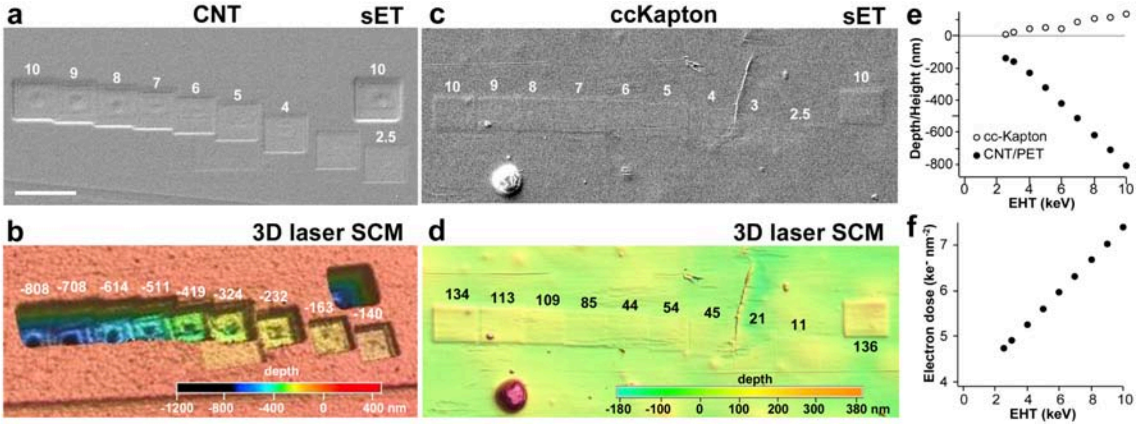

Supplementary Figure 3

SEM imaging causes beam damage on the CNT and cc-Kapton tape

a. Low magnification image captured with the sET detector shows a depression on the CNT tape surface by images captured with the BSD for 4096 x 4096 pixel image size, 6.4 μs dwell time, 3 nm pixel^{-1} with different acceleration voltages. Digits above the depressed square show the acceleration voltage value (keV) during imaging. Scale, 20 μm, is also for (c-d). **b**. The surface profile obtained with 3D laser SCM shows the depression depth clearly. Depression depth is indicated above the depression square. **c**. Low magnification image captured with the sET detector shows expansion of the cc-Kapton tape surface where the images were captured with the BSD. Digits above the expanded square show the acceleration voltage value (keV) during imaging. **d**. The surface profile obtained with 3D laser SCM shows the expansion height clearly. The expansion height is indicated above the depression square. **e**. Depression depth/expansion height is well correlated with acceleration voltage size. **f**. Electron dose of acceleration voltages.

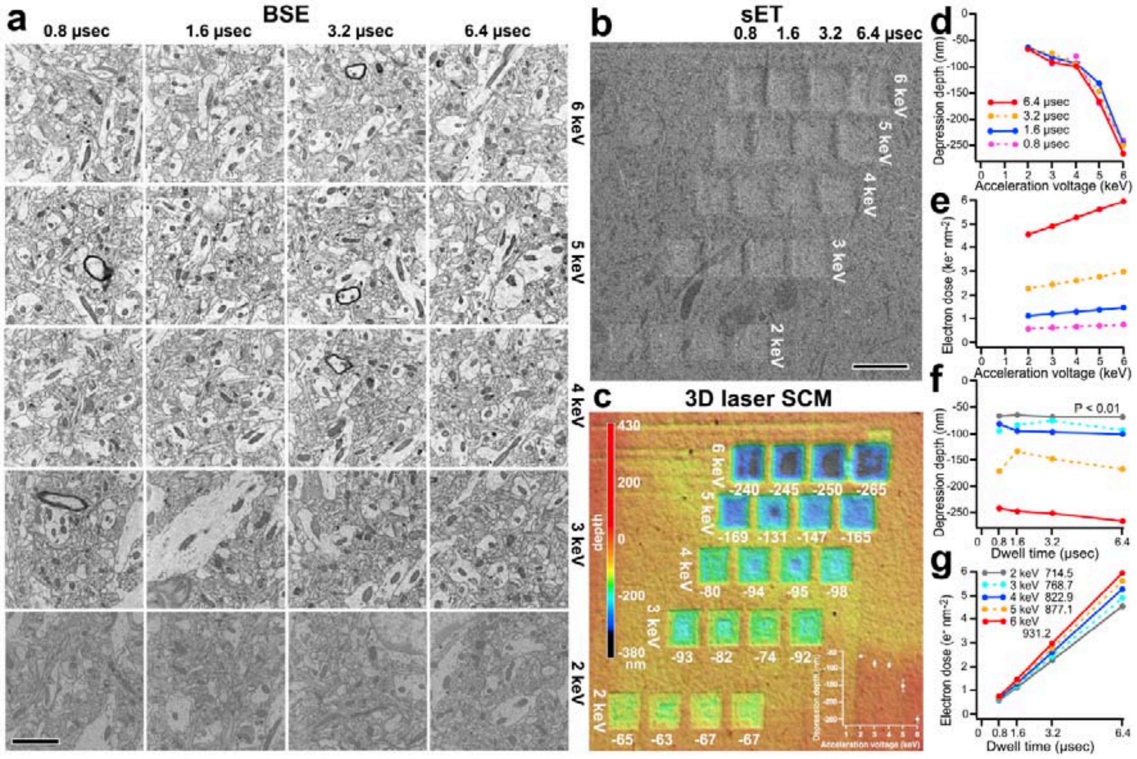

Supplementary Figure 4

Image quality varies with different acceleration voltage strengths and dwell times using a BSD with a single beam SEM.

a. Images of mHMS-treated brain tissue are captured with a BSD for 2048 x 2048 pixel image size, 3 nm pixel^{-1}, aperture 60 μm, with different acceleration voltage strengths (2 keV - 6 keV) and dwell times (0.8 μs - 6.4 μs) using an optimized working distance (7.6 mm: 6 keV, 7.7 mm: 5 keV and 4 keV, 7.8 mm: 3 keV, 7.9 mm: 2 keV) on the CNT tape. Scale, 2 μm. **b**. Low magnification image captured with the sET detector shows depression of the tissue section surface by images captured with the BSD with different acceleration voltages (2 - 6 keV) and dwell time (0.8, 1.6. 3.2, 6.4 μs). Scale, 10 μm, is also for (c). **c**. The surface profile obtained with 3D laser SCM shows the depression depth clearly. Measured depth (nm) is shown below each imaged square. Inlet graph at right bottom indicates averaged depression depth for each acceleration voltage. **d**. Depression depth at different acceleration voltages with different dwell times. **e**. Electron dose for each imaging condition. **f**. Depression depth at different dwell times with different acceleration voltages. **g**. Electron dose for each imaging condition. Electron dose rate (e^- nm^{-2} μs^{-1}) is shown at right of each acceleration voltage.

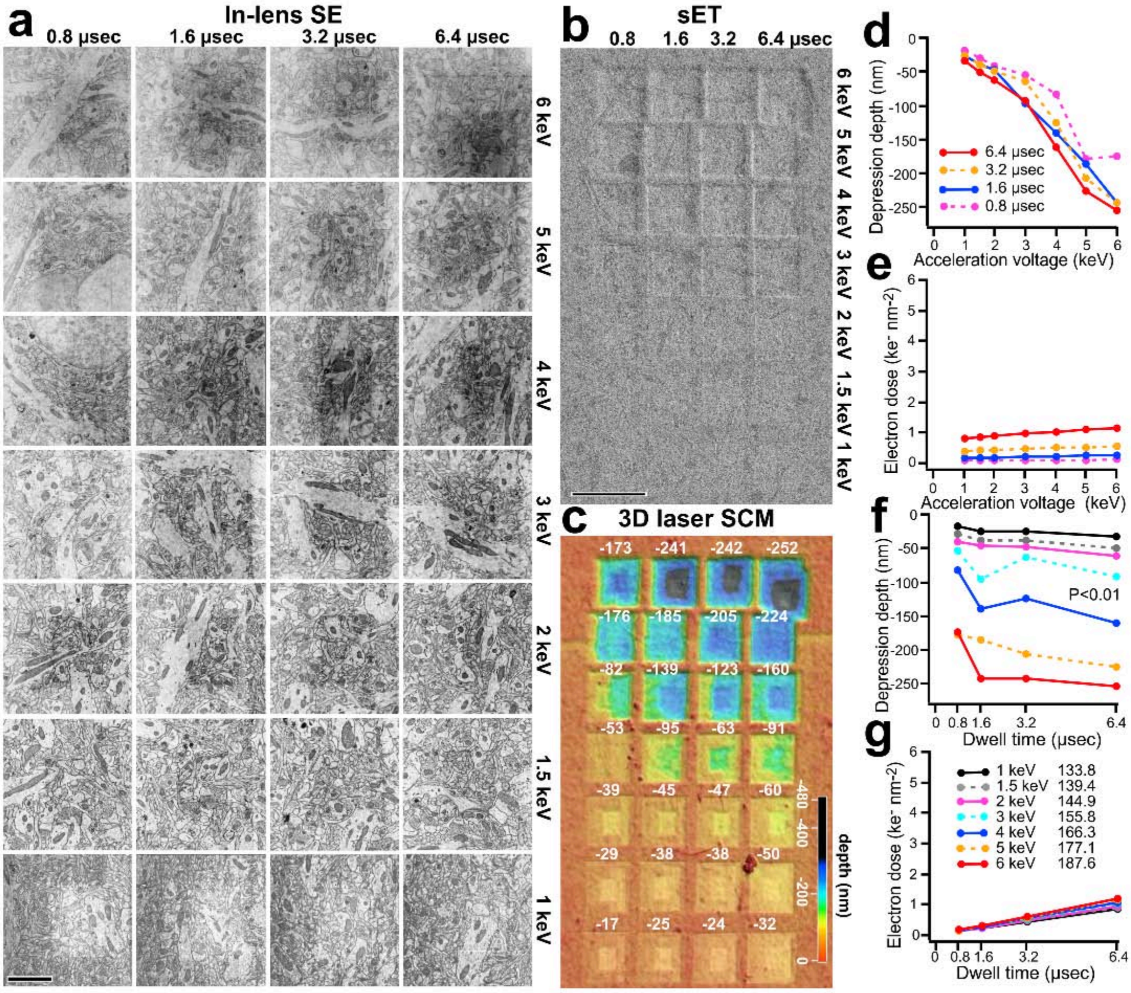

Supplementary Figure 5

Image quality varied with different acceleration voltage strengths and dwell times using an In-lens SE with a single beam SEM.

a. Images of mHMS-treated brain tissue are captured with an In-lens SE for 2048 x 2048 pixel image size, 3 nm pixel^{-1}, aperture 20 μm, with different acceleration voltage strengths (1 - 6 keV) and dwell times (0.8 μs - 6.4 μs) using an optimized working distance (4.0 mm: 6 keV - 4 keV, 4.1 mm: 3 keV - 1 keV) on the CNT tape. Some regions showing a darkening were focusing squares result of a thin layer of carbon adventitious build up, but not seen in 1.5 keV. In addition, a contrast reversal is apparent in the 1 kV images is seen. Scale, 2 μm. **b**. Low magnification image captured with the sET detector shows depression of the tissue section surface by images captured with the In-lens SE with the different acceleration voltages and the dwell time. Scale, 10 μm, is also for (c). **c**. The surface profile obtained with 3D laser SCM shows the depression depth clearly. **d**. Depression depth at different acceleration voltages with

different dwell times. **e**. Electron dose for each imaging condition. **f**. Depression depth at different dwell times with different acceleration voltages. **g**. Electron dose for each imaging condition. Electron dose rate (e^{-} nm^{-2} μs^{-1}) is shown at right of each acceleration voltage.

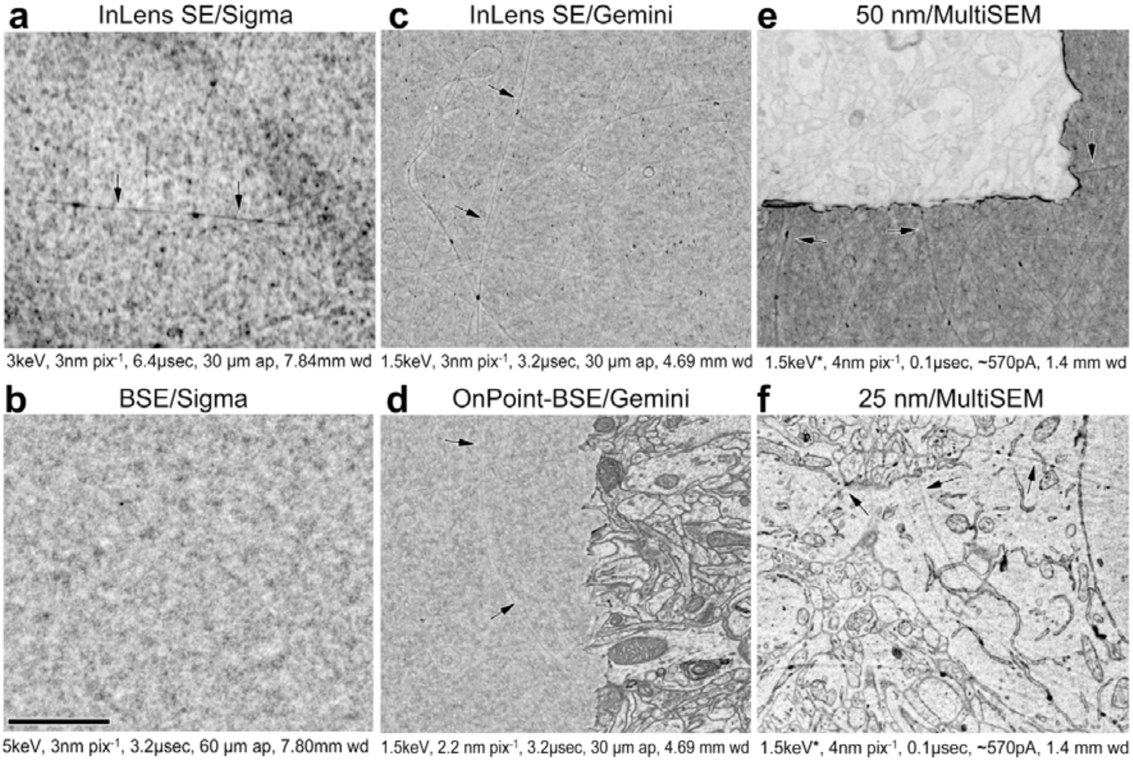

Supplementary Figure 6

The CNT tape surface is almost uniform and CNTs are observed and transparently seen under some imaging conditions.

a. Surface structure of the CNT tape imaged with an In-lens SE detector in Sigma SEM. Only thick CNTs are observed (arrows). **b**. Surface structure of the CNT tape imaged with a BSD in Sigma SEM. **c**. Surface structure of the CNT tape imaged using In-lens SE detector with the stage bias potential in Gemini SEM. Many CNTs are observed (arrows). **d**. Surface structure of the CNT tape (left half) and brain tissue image from a 50 nm thick section on the tape (right half) captured with OnPoint-BSD in Gemini SEM. CNTs are observed in low contrast (arrows). **e**. Surface structure of the CNT tape (bottom half) and brain tissue image from a 50 nm thick section on the tape (upper left) captured with MultiSEM. CNTs are observed only on the surface of the tape (arrows). **f**. Brain tissue image from a 25 nm thick section prepared according to Hua et al. [9] collected on CNT tape and captured with a 61 beam MultiSEM. CNTs are translucently observed in the tissue (arrows). Scale in (**b**), 2 μm, is also for (**a, c-f**).

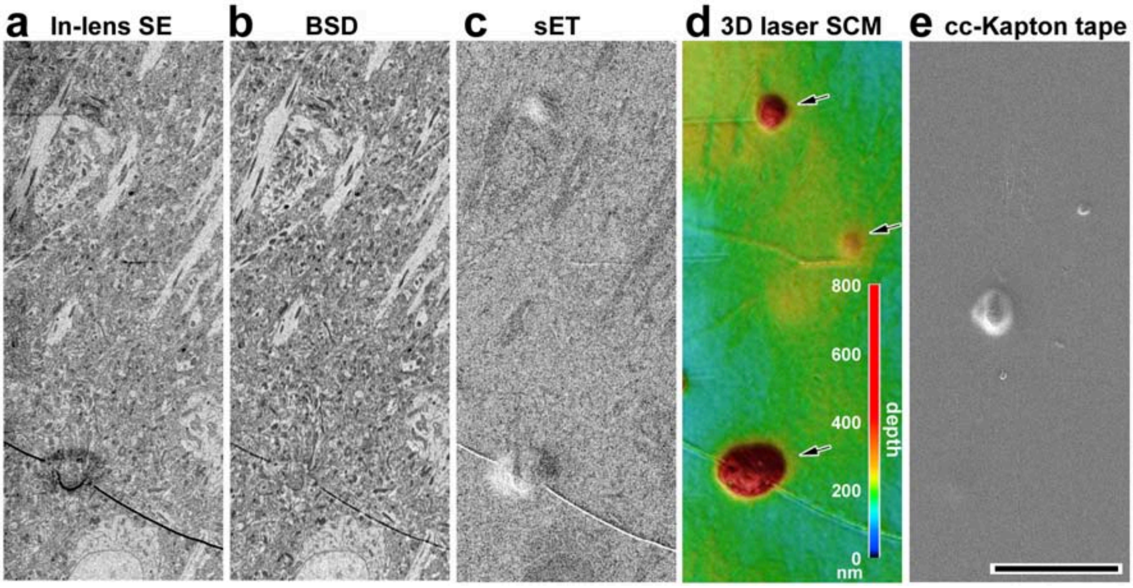

Supplementary Figure 7

Surface irregularities of the cc-Kapton tape have a slightly adverse effect on image quality.

a. Tissue image captured with In-lens SE detector, **b**. with BSD, **c**. with sET, **d**. with 3D laser SCM. Some part of the tissue section show bumps presumably caused by small carbon particle balls (arrows in d), which are seen in image obtained with In-lens SE detector (**a**) and sET (**c**), but not with BSD (**b**). **e**. Bumps in the different part of the cc-Kapton tape captured with sET. Scale in (**e**), 10 μm, is also for (**a-d**)).

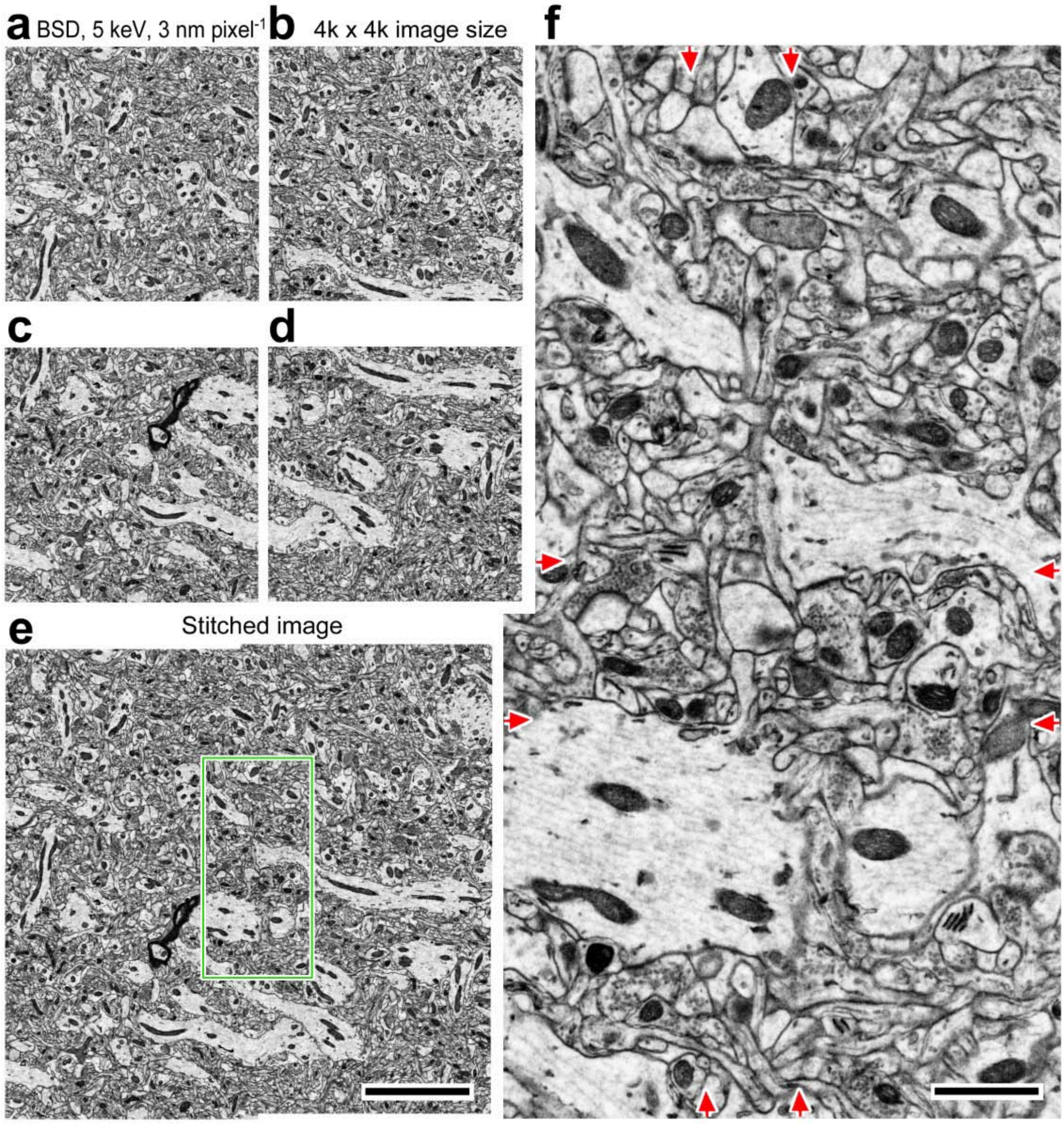

Supplementary Figure 8

Stitching image tiles can be done without any noticeable gap or seam.

a-d. Tile images (2 x 2) of the mHMS ultrathin section of cortex captured with BSD for 3.2 μs dwell time, 3 nm pixel^{-1}, 60 nm aperture at 5 keV, 2.8 ke^{-}nm^{-2} electron. **e.** Stitched tiles. Scale, 5 μm, is for (**a-d**). **f.** Enlarged image of rectangle in e shows possible seams (red arrows). No noticeable seams are observed. Scale. 1 μm.

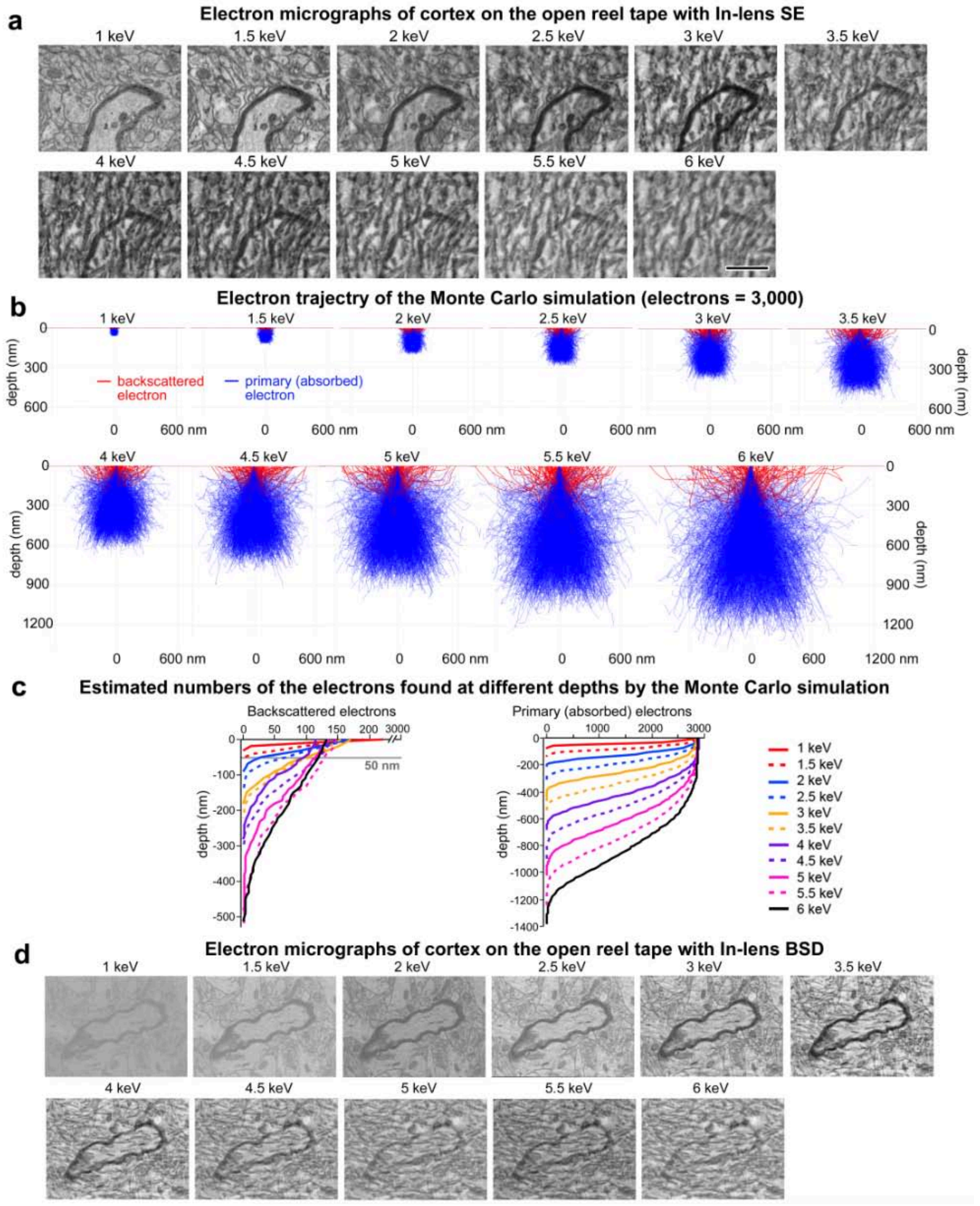

Supplementary Figure 9

Interaction volume of primary and BSEs for different acceleration voltages can be estimated with electron tracks by Monte Carlo simulation

a. Image of an ultrathin section of mHMS-treated brain tissue on open reel tape captured with In-lens SE detector at different acceleration voltages (**a**. 1 keV - **k**. 6 keV). Scale, 1 μm, is also for (**d**). **b.** Monte Carlo simulation. Blue lines show primary

(absorbed) electron tracks and red lines show BSE tracks with different acceleration voltages. **c.** Estimated numbers of the electrons in different acceleration voltages found at different depth. **d.** Image of an ultrathin section of mHMS-treated brain tissue on open reel tape captured with BSD at different acceleration voltages (**a**. 1 keV - **k**. 6 keV). BSE: backscattered electron, PE: primary electron.

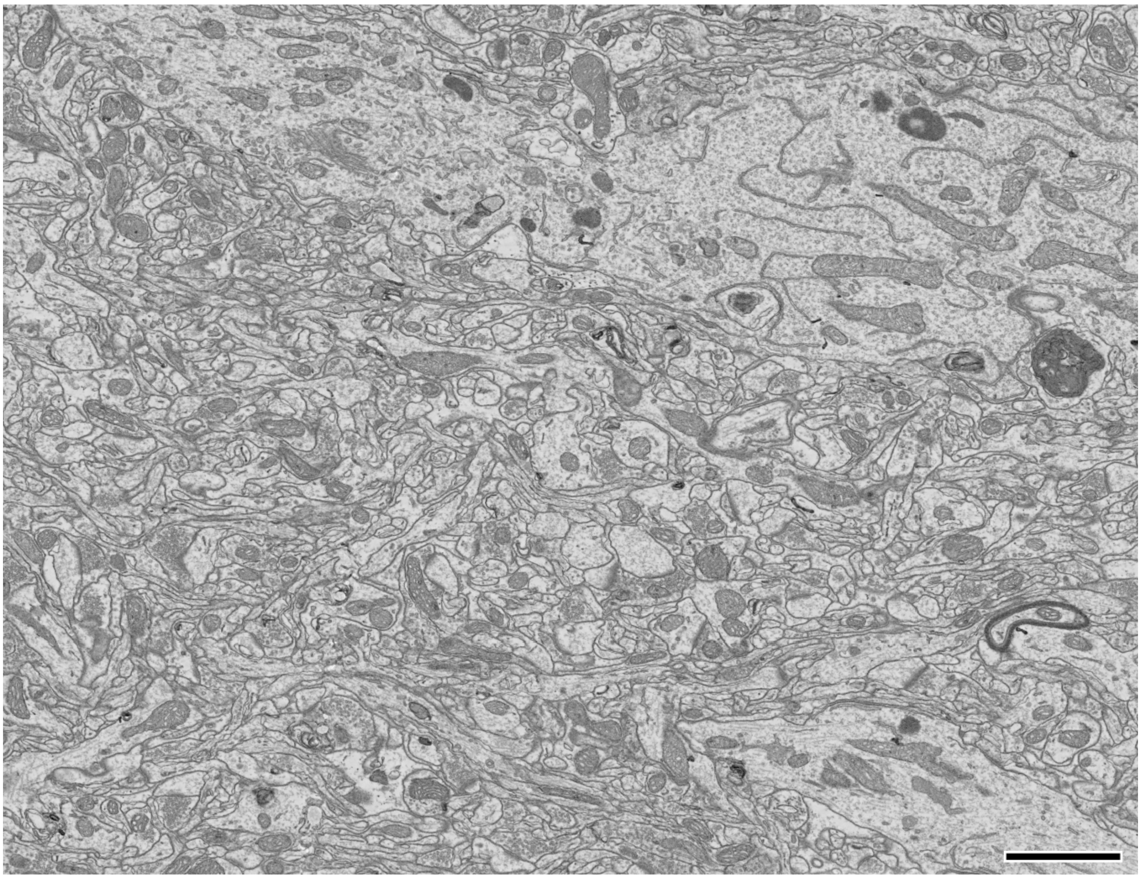

Supplementary Figure 10

High-resolution brain tissue image made with the TOLA protocol and lead citrate section staining captured using a BSD optimized for low accelerating voltage (OnPoint BSD, Gatan Inc.) with 3.2 μs dwell time, 1 nm pixel^{-1}, 5 mm working distance, 30 μm aperture at 1.5 keV. Scale, 2 μm.

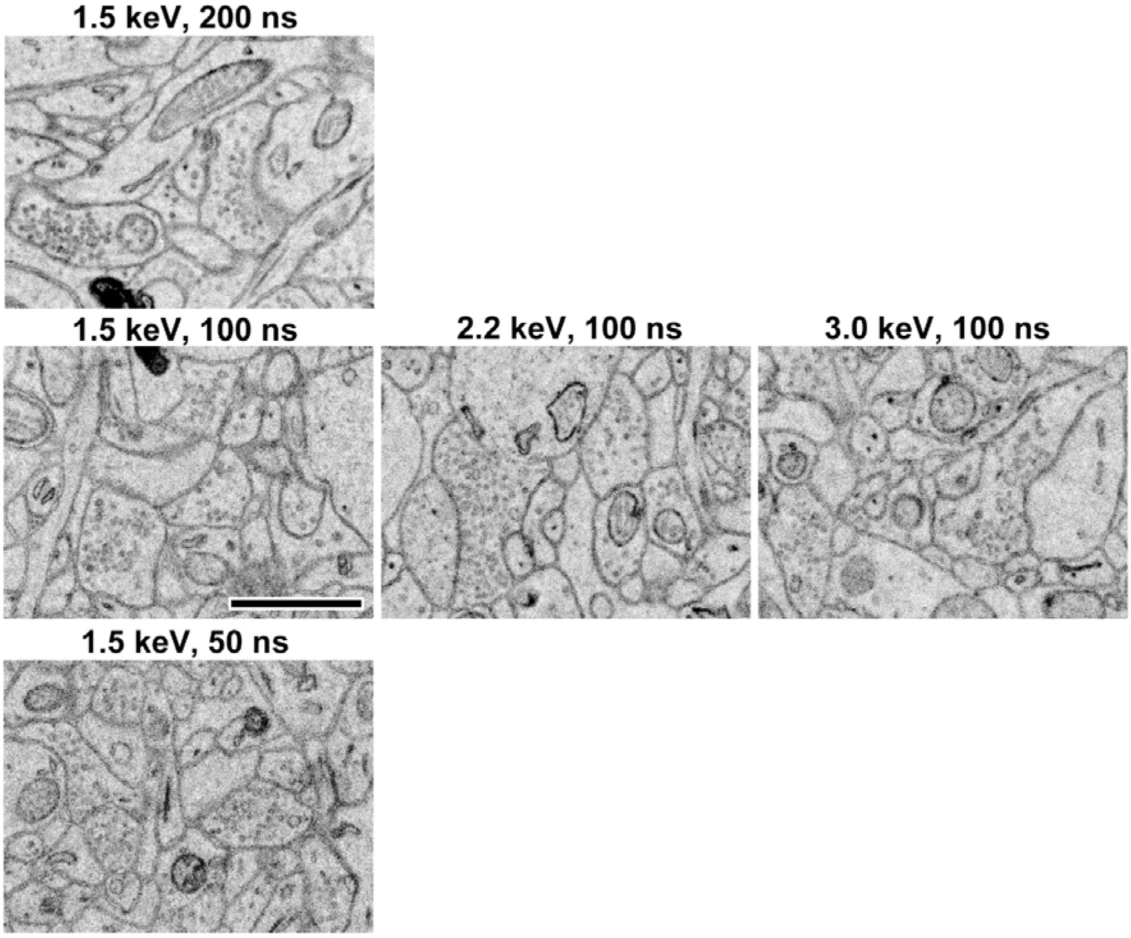

Supplementary Figure 11

Image quality varies with different acceleration voltage strengths and dwell times using a MultiSEM.

The images were taken from a 35 nm-thick section of *en-bloc* stained cortical mouse brain tissue at a pixel size of 4 nm. Scale, 1 μm.

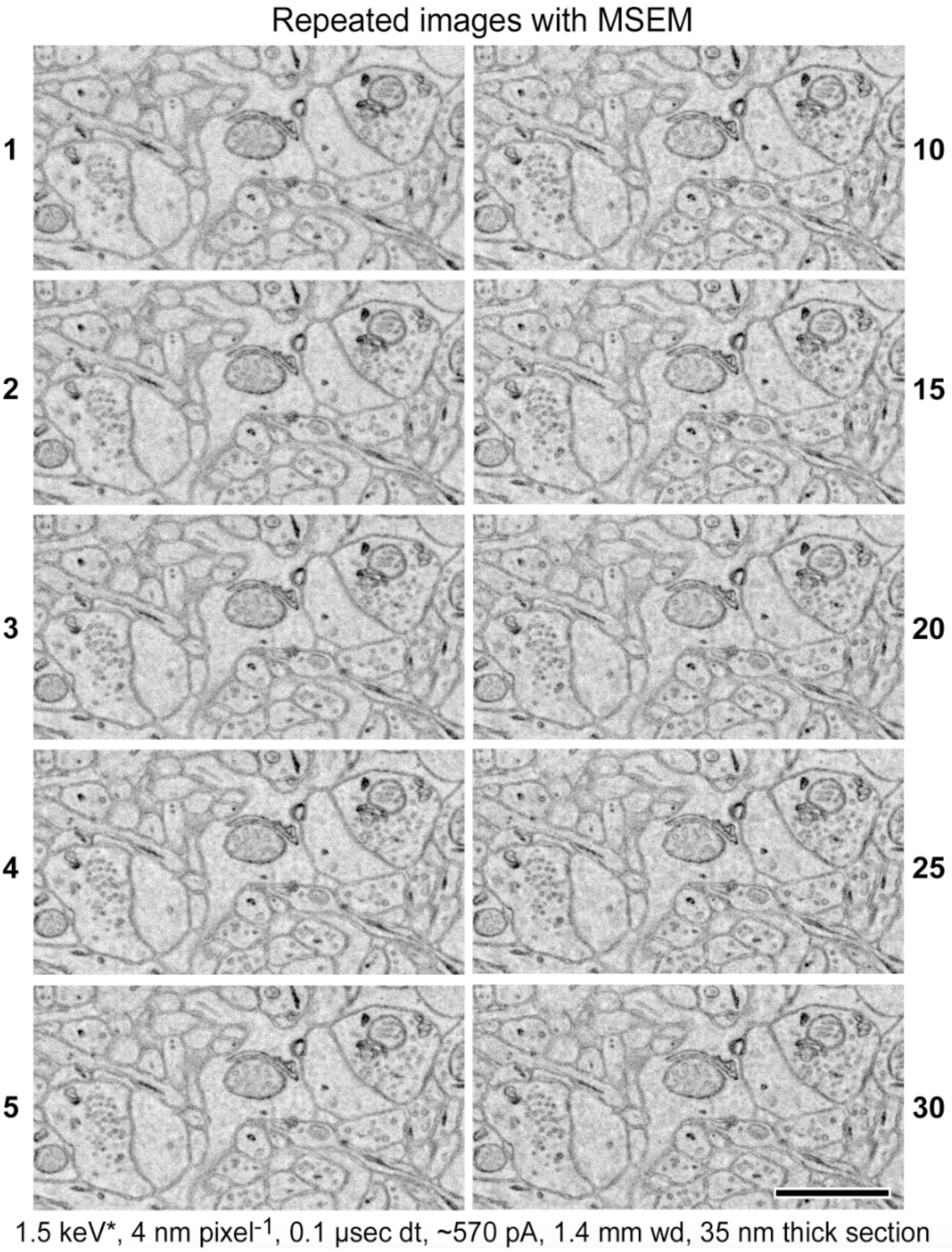

Supplementary Figure 12

Multiple exposures in the MultiSEM do not greatly affect image quality

The images were taken from a 35 nm-thick section of *en-bloc* stained cortical mouse brain tissue using an acceleration voltage of 1.5 keV, a pixel size of 4 nm and a pixel dwell time of 100 ns. The number on the side of the image indicates instances of repeated imaging. Scale, 1 μm. wd, working distance; dt, dwell time.

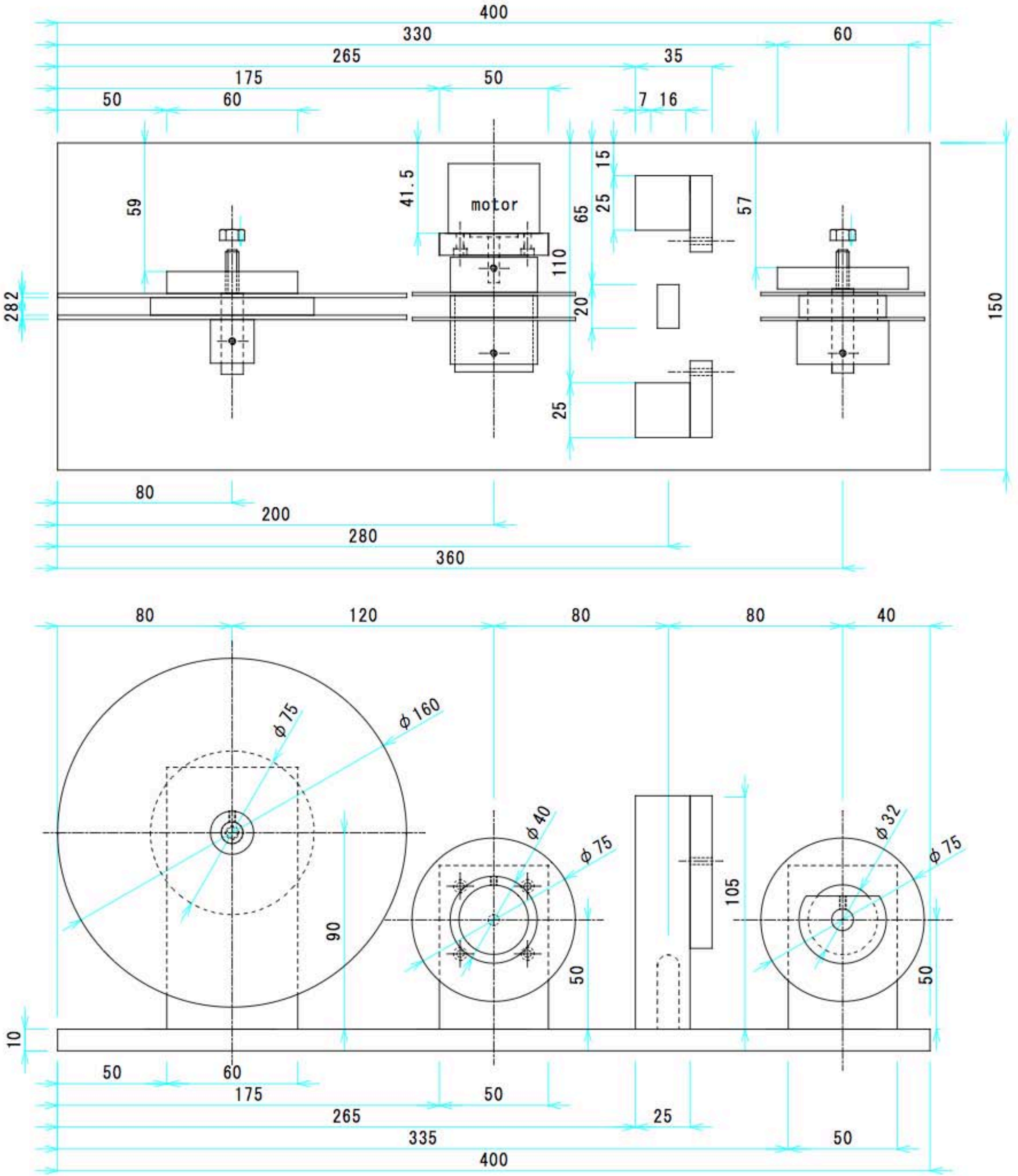

Supplementary Figure 13

Sketch of the reel-to-reel motorized winder for plasma discharge treatment. The unit of the numerical value is mm.

Supplementary Tables

Supplementary Table 1 Specification of Tapes

Tape material	Coated material	Company	Thickness (μm)	Commercial product			Sheet resistance (Ω □$^{-1}$)	Total light transmission (%)	URL
				Tape (8mm width)	Film	Product name			
Kapton HN	none	DuPont	50	n/a	available	Kapton HN	1x10^{16}	72.2	http://www.dupont.com/products-and-services/membranes-films/polyimide-films/brands/kapton-polyimide-film.html/
	carbon	RMC Boeckeler	50	available	none		19.2/107 6,530x10^{6}	47.9	http://www.rmcboeckeler.com
	germanium	Sheldahl	50	n/a	available	IP# C903907-1	n/a	none	http://www.sheldahl.com/default.aspx
Copper foil	none	Takeuchi Metal Foil & Powder	20	n/a	available		0.1	none	http://www.etakeuchi.co.jp
			40	n/a	available		0.1	none	
Open reel	magnetic crystals	EMG International	50	available	n/a	Studio Master SM911 1/4 inch width	n/a	none	www.rmgi.nl http://www.recordingdataservice.com/default.asp
PET	ITO	TDK	53	n/a	available	Fleclear	155	90.1	http://www.tdk.co.jp/index.htm
		Teijin	50	n/a	available	Eleclear	150	89.6	https://www.teijin.com
	CNT	Toray	50	n/a	not on sale		240/500	88.4	http://www.toray.com
		Teijin	50	n/a	not on sale		800/1,000	86.2	https://www.teijin.com, commercially available by RMC in 2018

Supplementary Table 2 Depth of the electrons reached and signal efficiency for a 50 nm thick section

Acceleration voltage		1	1.5	2	2.5	3	3.5	4	4.5	5	5.5	6
DER (nm)	BSE	-25	-53	-89	-130	-173	-217	-274	-293	-474	-517	-504
	PE	-70	-137	-206	-301	-467	-570	-763	-897	-1016	1231	-1375
SEF50 (%)		100.0	99.5	83.0	57.4	40.4	30.3	15.7	18.9	14.8	12.8	9.8

DER: Depth of the electrons reached; BSE: backscattered electron; PE: Primary electron
SEF50: Signal efficiency for a 50 nm thick section

Supplementary Table 3 Michelson contrast and Contrast-to-noise ratio

Detector	BSE					
Accerelation voltage (keV)	2	3	4	5	6	7
Michelson contrast	0.277	0.374	0.376	0.272	0.302	0.273
Contrast-to-noise ratio	0.144	0.209	0.208	0.211	0.199	0.193
Detector	In-Lens SE					
Accerelation voltage (keV)	1	1.5	2	3	4	5
Michelson contrast	0.156	0.231	0.246	0.211	0.180	0.168
Contrast-to-noise ratio	0.145	0.202	0.219	0.195	0.161	0.141

Supplementary References

1 Hayworth, K. J., Kasthuri, N., Schalek, R. & Lichtman, J. W. Automating the collection of ultrathin serial sections for large volume TEM reconstructions. *Microsc Microanal* **13**, 86-87 (2006).

2 Kasthuri, N. *et al*. Saturated Reconstruction of a Volume of Neocortex. *Cell* **162**, 648-661 (2015).

3 Mikuni, T., Nishiyama, J., Sun, Y., Kamasawa, N. & Yasuda, R. High-Throughput, High-Resolution Mapping of Protein Localization in Mammalian Brain by In Vivo Genome Editing. *Cell* **165**, 1803-1817 (2016).

4 Drouin, D. *et al*. CASINO V2.48: a fast and easy-to-use modeling tool for scanning electron microscopy and microanalysis users. *Scanning* **29**, 92-101 (2007).

5 Reimer, L. *Image Formation in Low-Voltage Scanning Electron Microscopy*. 2-3 (SPIE Press, 1993).

6 Scipioni, R. *et al*. Electron microscopy investigations of changes in morphology and conductivity of LiFePO4/C electrodes. *J Power Sources* **307**, 259-269 (2016).

7 Bouwer, J. C. *et al*. Deceleration of probe beam by stage bias potential improves resolution of serial block-face scanning electron microscopic images. *Adv Struct Chem Imaging* **2**, 11 (2017).

8 Ohta, K. *et al*. Beam deceleration for block-face scanning electron microscopy of embedded biological tissue. *Micron* **43**, 612-620 (2012).

9 Hua, Y., Laserstein, P. & Helmstaedter, M. Large-volume *en-bloc* staining for electron microscopy-based connectomics. *Nat Commun* **6**, 7923 (2015).
