# Supplementary material for: A carbon nanotube tape for serial-section electron microscopy of brain ultrastructure
Source: Nat Commun. 2018 Jan 30;9:437. doi: 10.1038/s41467-017-02768-7 (PMC5789869; doi:10.1038/s41467-017-02768-7)
Supplement: Supplementary file 3 — Description of Additional Supplementary Files [file 41467_2017_2768_MOESM3_ESM.pdf]

Description of Supplementary Files

File Name: Supplementary Movie 1
Description: Serial ultrathin section images with GABA post-embedding immunoreaction stained with 15nm colloidal gold particles of rat frontal cortex.
